# Early mobilization on continuous renal replacement therapy is safe and may improve filter life

**DOI:** 10.1186/s13054-015-0781-4

**Published:** 2015-04-28

**Authors:** Frederic M Jacobs

**Affiliations:** Service de Réanimation Polyvalente, Hopital Antoine Béclère AP-HP, 157 rue de la Porte de Trivaux, Clamart, 92140 France

In their study Wang and colleagues [1] found that mobilization during continuous renal replacement therapy (CRRT) is safe, and did not lead to filter circuit clotting. However, I feel that this work raises two remarks.

First, by delivering CRRT via continuous venovenous hemodiafiltration (CVVHDF) with a dialysate rate of 20 ml/kg/hour, a replacement fluid rate of 15 ml/kg/hour and a low effluent fluid removal rate, the filtration fraction, a major determinant of clotting, may have been lower than the one resulting from continuous venovenous hemofiltration (CVVHF), which is as much used as the CRRT mode [2]. Furthermore, details concerning the blood flow (which impacts filtration fraction) are lacking. Thus, generalization of these results should probably be tempered.

Finally, regarding the exclusion criteria, the use of intermittent hemodialysis may be the best way to ensure daily patient mobilization, at a much lower cost [3].

## Abbreviations

CRRT: Continuous renal replacement therapy
CVVHDF: Continuous venovenous haemodiafiltration
CVVHF: Continuous venovenous haemofiltration
