# Supplementary material for: Early mobilization on continuous renal replacement therapy is safe and may improve filter life
Source: Crit Care. 2015 Apr 28;19(1):205. doi: 10.1186/s13054-015-0781-4 (PMC4411787; doi:10.1186/s13054-015-0781-4)
Supplement: Related texts

# Reply: Authors’ response

**Authors:** Yi Tian Wang, Nigel Fealy, Terry P Haines, Elizabeth H Skinner

We read the letter by Jacobs with interest. We agree that a combined dose of 35 ml/kg/hour (incorporating both convective and diffusive clearance) in CVVHDF mode would reduce filtration fraction compared with a pure convective mode such as CVVHF. However, there is no conclusive evidence that filter life is related to filtration fraction, while there is some evidence to the contrary [4]. Our original manuscript agreed that the generalizability of the results should be tempered and stated that we felt large multi-center studies were warranted to confirm the findings [1]. We agree that blood flow rates in the circuit should be collected in such studies, as this may be an independent confounding factor for circuit life and filtration fraction. However, we conducted an additional *post hoc* sensitivity analysis adjusting for blood flow and the effect of the MOVE (movement on vascular catheter evaluation) intervention on filter life was still significant in our cohort (regression co-efficient (robust 95% CI), *P* value = 14.3 hours (5.3, 23.3), *P* = 0.003).

Moreover, mobilization restrictions placed on patients undergoing CVVHDF via femoral vascath frequently preclude the delivery of evidence-based practice [5, 6], where early mobilization reduces ventilation duration and ICU and hospital length of stay [7]. Whilst intermittent hemodialysis is an alternative method of CRRT delivery, CVVHDF is the therapy of choice in over 50% of Australian ICUs (unpublished data). The aim of our study was to test whether patients subjected to continuous therapies via femoral access could be safely mobilized. We reiterate our conclusions, which were that mobilization of patients undergoing CRRT via femoral vascular access was safe and feasible [1].
